# In Situ IgM Production and Clonal Expansion of B-1 Cells in Peritoneal Cavity Promote Elimination of *C. albicans* Infection in IgH Transgenic Mice with V_H_ Derived from a Natural Antibody

**DOI:** 10.1371/journal.pone.0060779

**Published:** 2013-04-02

**Authors:** Rong Tian, Meng Fu, Zhuo Zhang, Jing Ren, Jingang An, Yufeng Liu, Wei Li

**Affiliations:** 1 Department of Dermatology, General Hospital of the Air Force, Beijing, P. R. China; 2 Department of Dermatology, Xijing Hospital, Xi’an, P. R. China; 3 Department of Neurology, Tangdu Hospital, Xi’an, P. R. China; Fudan University, China

## Abstract

B-1 cells are innate-like cells that play important roles in host defense against infection. However, the function of B-1 cells in fungi infection remains unclear. Previously we produced IgH transgenic mice TgV_H_3B4 with V_H_ derived from a natural antibody 3B4 that can identify *C. albicans*, and found that TgV_H_3B4 mice were resistant to intraperitoneal (i. p.) and intravenous *C. albicans* infection. Most of the peritoneal cavity (PEC) B-1 cells in TgV_H_3B4 mice express transgenic BCR that binds *C. albicans*. In the present study, we explored the response of B-1 cells to *C. albicans* infection by applying i. p. inoculation of fungi in TgV_H_3B4 mice. We found that *C. albicans* was cleared more efficiently in TgV_H_3B4 mice after i. p. inoculation than that of littermate control. The level of *C. albicans*-reactive IgM in PEC of TgV_H_3B4 mice was much higher than that of control, and the number of B-1a B cells was also elevated in TgV_H_3B4 mice, which was mainly due to enhanced proliferation of B-1 cells. Additionally, numbers of *C. albicans*-specific B cells increased greatly in TgV_H_3B4 mice after *C. albicans* inoculation. Our data suggested that in situ IgM production and clonal expansion of B-1 cells in PEC participate in host defense against *C. albicans* infection.

## Introduction

Natural antibodies, major component of the innate humoral immunity [1], [2], have been shown to be important in innate and adaptive immune responses against microbial infections [3]–[7]. The poly-reactive natural IgM and surface antigen receptor expressed by B-1 cells might be a kind of pattern recognition receptors [8], and can recognize invariant molecular structures shared by large groups of pathogens, including many fungi [8]–[10]. The protective role of natural antibodies against bacteria and virus infection has long been recognized [4], [6], however, the importance of natural antibodies in host defense against fungi infection has not been revealed untill recently. B cell-depleted mice have been shown to be more susceptible to systemic candidiasis than controls [11], and B cell knockout mice are easier to develop systemic, but not mucosal, candidiasis [12]. More recently, Kozel's group showed that a mannan-specific IgG antibody from normal human mediates C3-binding and killing of *C. albicans* [13], [14]. We produced a monoclonal polyreactive natural antibody [8], [15], 3B4, which not only recognizes self-antigen keratin, but also recognizes a surface antigen located in the germ tubes of *C. albicans*, and protects mice from *C. albicans*-induced death with passive immunization, by mechanisms involving suppression of germ tube formation and modulation of phagocytosis [8]. Using the V_H_ gene from 3B4, we constructed a μ chain transgenic mouse (TgV_H_3B4) which has elevated serum anti-*C. albicans* IgM and is resistant to *C. albicans* infections. Analyses of B cell development showed that in TgV_H_3B4, most B cells secreting *C. albicans*-reactive antibodies are selected into the B-1 B cell compartment [8]. These results demonstrate a key role of natural antibodies in anti-*C. albicans* immune responses, and also indicate that B-1 cells may be important in anti-fungi immunity.

B-1 cells are distinguished from other subsets of B cells by their anatomical localization, phenotype, self-renewing capacity, and production of natural antibodies [16], [17]. B-1 cells constitute a major fraction of B cells present in peritoneal cavity (PEC), and are a minor fraction of B cells in spleen [16], [17]. Based on cell surface CD5 expression, B-1 cells are subdivided into the B-1a (CD5^+^) and B-1b (CD5^−^) subsets, and B-1 cells in PEC also express Mac-1. Although the origin of B-1 cells is still in debate, there is evidence indicating that B-1 cells are positively selected by self-antigens, and strong BCR antigen signals appear to be important for the decision to become B-1 cells [16], [18], [19]. B-1 cells are characterized by the production of natural antibodies in the absence of apparent infection or immunization [16], and B-1-specific antibodies are involved in many biological events, such as reducing atherosclerotic lesions, activating T cell responses, contributing to autoimmunity, and promoting ischemia/reperfusion injury [20]–[22]. Most importantly, B-1 cells are envisioned as key players in the early humoral response against pathogens and are thought to be the primary antibody producers in response to T cell-independent type 2 (TI-2) antigens along with marginal zone B cells [23]–[25]. However, the role of B-1 cells in fungal infection and how B-1 cells respond to pathogen infection in peritoneal cavity are still not clear.

In the present study, intraperitoneal (i. p.) *C. albicans* inoculation was applied to our transgenic model of TgV_H_3B4, in which *C. albicans*-reactive B-1 cells were enriched in PEC, to explore the role and immune response of B-1 cells in fungi infection. Our data showed that *C. albicans*-specific B-1 cells proliferated actively and produced a large amount of natural IgM to clear the pathogen, indicating that B-1 cells play a critical role in host defense against *C. albicans* infection.

## Results

### Efficient clearance of *C. albicans* in PEC of TgVH3B4

In previous study, we found TgV_H_3B4 mice were resistant to both intravenous (i. v.) and i. p. *C. albicans* infection. In particular, almost complete protection was observed in i. p. inoculation with a relatively high number of fungi in TgV_H_3B4 mice [8]. It seemed likely that local environment of PEC might exert more efficient defense against *C. albicans* infection. In this study, we set out to analyze in detail the antibody and B-1 cell responses in PEC after i. p. *C. albicans* inoculation. A lower dose of *C. albicans* (2×10^6^) was applied for i. p. inoculation, and our previous study has shown that the infection would be restricted in PEC and all the mice would survive.

**Figure 1:**
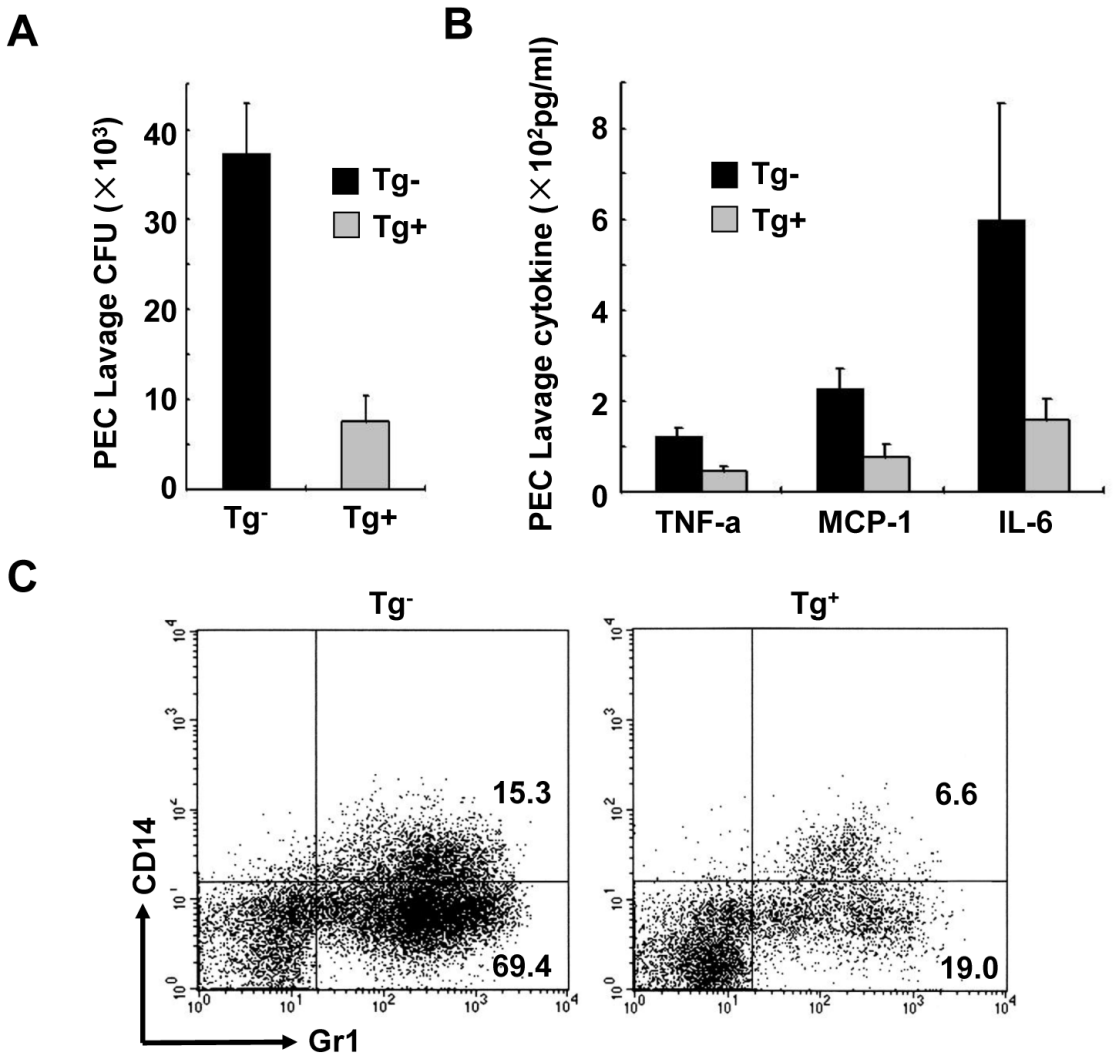
*C. albicans* burden and inflammatory infiltration after i. p. inoculation. 2×10^6^ *C. albicans* yeasts were i. p. injected into TgV_H_3B4 mice and littermate. At different time point the mice were sacrificed and PEC lavage was eluted by PBS. Results obtained from elution at 36 h after the inoculation were shown. Living yeasts in the elution were assessed by a colony-forming assay (A), and supernatant of the elution was subjected to inflammation cytokines analysis by a Cytometry Beads Array kit (B). Data shown are means±SD, n = 6. * *P*<0.01. (C) Cells collected from the PEC elution were stained with antibodies and analyzed by FCM. Data are representative of four independent experiments.

The *C. albicans* burden and inflammatory infiltration in PEC after fungi inoculation were analyzed in TgV_H_3B4 mice and littermate control. At different time point after *C. albicans* inoculation, the lavages were eluted from the mice. The supernatant of the elution was subjected to inflammatory cytokine analysis, and the cells in the elution were subjected for colony-forming assay and FCM analysis. The colony-forming units (CFU) of the elution after *C. albicans* inoculation decreased with time, and level of inflammatory cytokines and neutrophil infiltration increased gradually and began to decrease from 36h after inoculation. The data at 36 h after inoculation were shown in Fig. 1. The number of living yeasts in the lavage of TgV_H_3B4 mice was much lower than that of control (Fig. 1A). The concentrations of inflammatory cytokines, TNF-a, IL-6 and MCP-1, were approximately 3 folds lower in TgV_H_3B4 mice than that of their littermates (Fig. 1B). There were fewer neutrophils in the PEC lavage of TgV_H_3B4 mice when analyzed using anti-Gr1 antibody (Fig. 1C). These data showed that there were less *C. albicans* burden, less neutrophil infiltration and inflammatory cytokines in PEC of TgV_H_3B4 mice, indicating that *C. albicans* was cleared more efficiently in TgV_H_3B4 mice.

### Elevated IgM production in PEC of TgVH3B4

B-1 cells are the major source of natural IgM antibodies which constitute the first line of defense against infection. In the TgV_H_3B4 mice, most B cells in PEC produce the 3B4 IgM that can recognize *C. albicans* [8], [26]. Our previous study have demonstrated that the 3B4 antibody protected mice from *C. albicans*-induced death in passive immunization, by mechanisms involving suppressing the germ tube formation and modulating phagocytosis. Thus, to elucidate the mechanism for the enhanced clearance of *C. albicans* in TgV_H_3B4 mice, we first analyzed the antibodies production in PEC before and after fungi inoculation.

**Figure 2:**
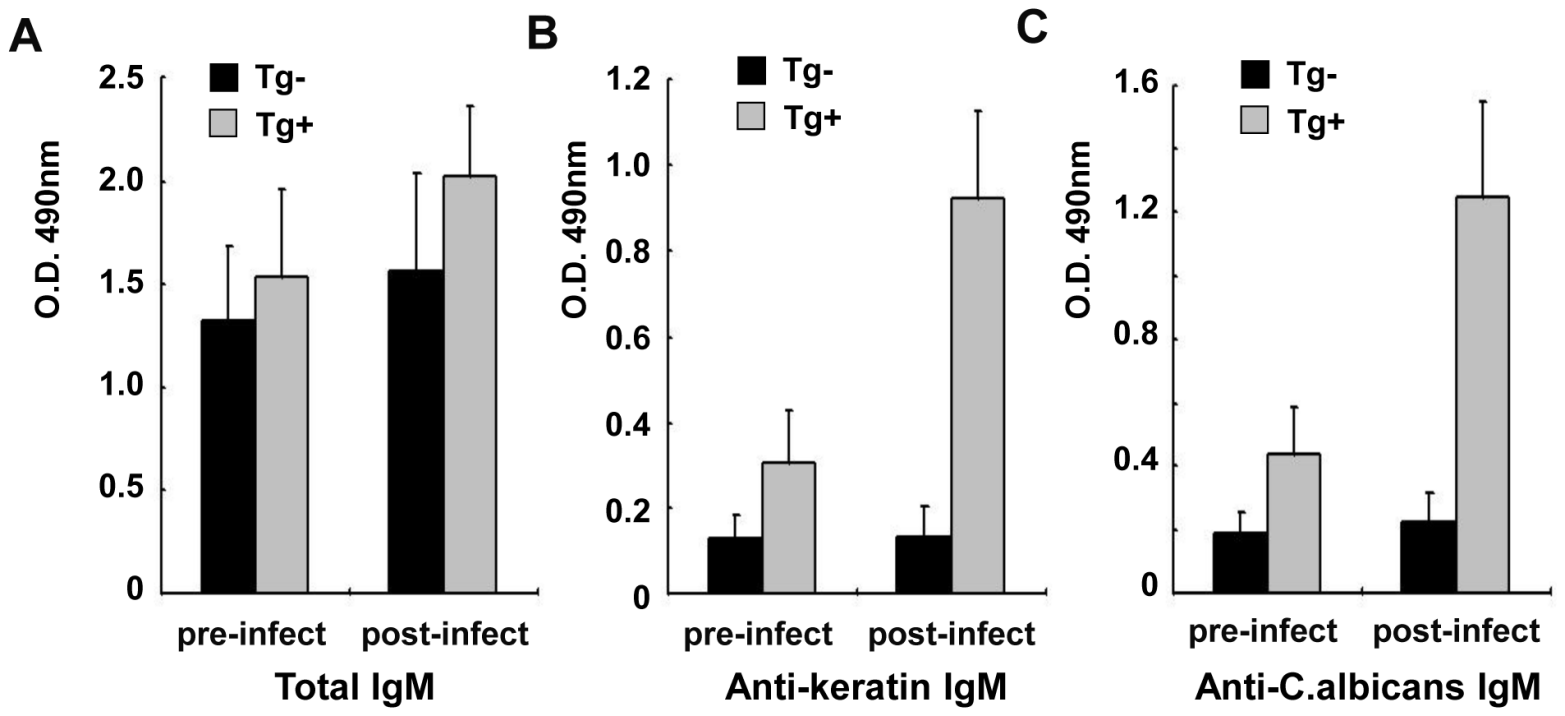
Natural IgM production in PEC. PEC lavage before and after *C. albicans* inoculation were detected by ELISA using plate coated with anti-mouse IgM antibody or live *C. albicans*. (A) Total IgM, (B) *C.albicans*-specific IgM. Data shown are means±SD, n = 6. * *P*<0.01.

Before fungi inoculation the total IgM level was almost the same between TgV_H_3B4 mice and control, however, the level of *C. albicans*-specific IgM in PEC of TgV_H_3B4 mice was much higher than that of control (Fig. 2A-B). After *C. albicans* inoculation, the total IgM level increased slightly in both TgV_H_3B4 and control mice, as shown in Fig. 2A. However, the level of *C. albicans*-specific IgM increased greatly in TgV_H_3B4 mice, in contrast to a slight increase of anti-*C. albicans* IgM in the control mice (Fig. 2B). There was no difference of total and *C. albicans*-specific IgG antibodies in PEC elution before and after inoculation between TgV_H_3B4 mice and control (data not shown). These data indicated that B-1 cells produce natural IgM in PEC at a baseline level in normal conditions, and are stimulated to produce larger amount of antigen-specific antibodies upon pathogen inoculation.

### Increased proliferation of B-1 cells after *C. albicans* inoculation

**Figure 3:**
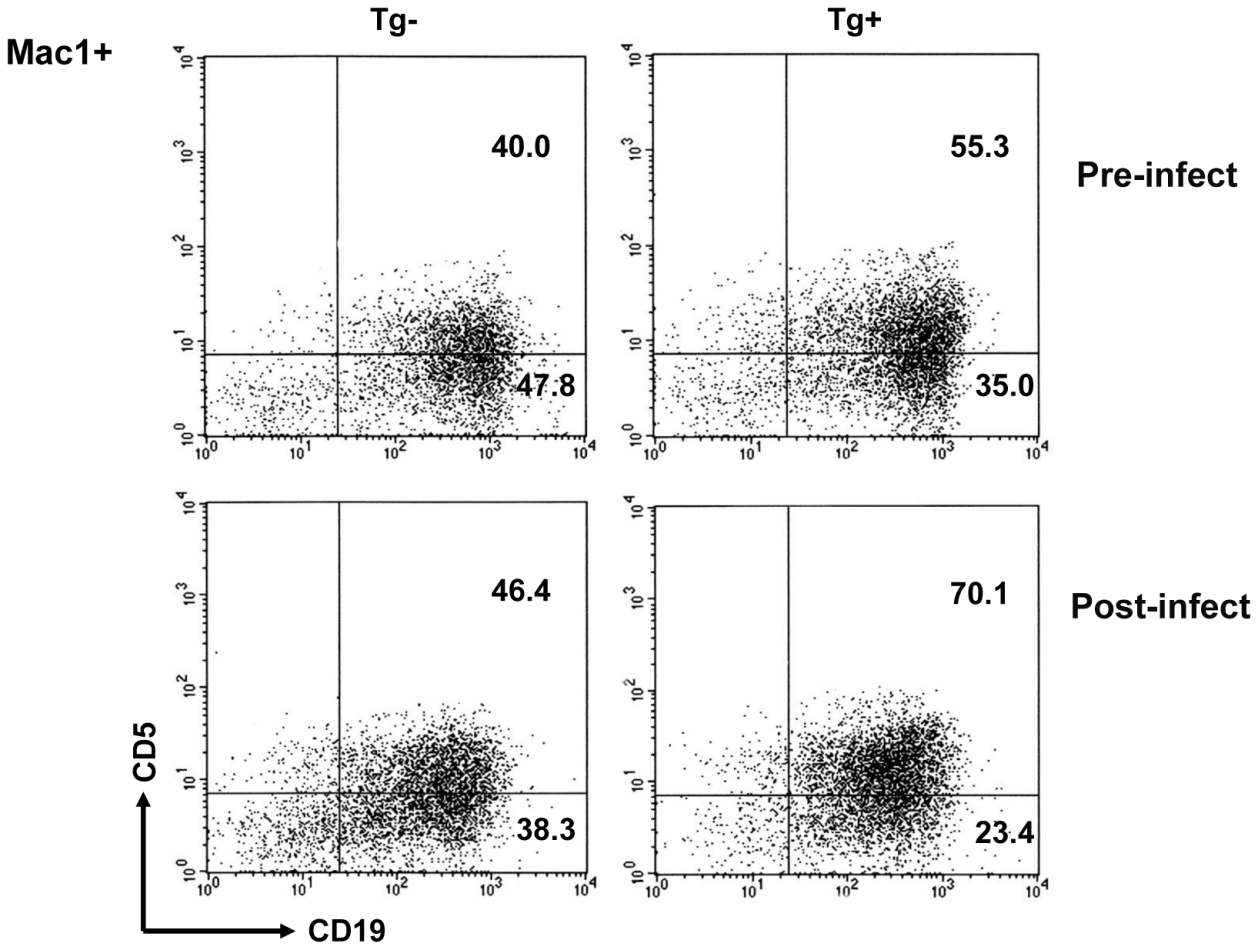
B cell analysis in PEC. Cells collected the PEC lavage before and after *C.albicans* inoculation were stained with antibodies and analyzed by FCM. Data are representative of four independent experiments.

As B-1 cells are the major producing cells of natural antibodies, we analyzed B-1 cells' number and proliferation in PEC after *C. albicans* infection. Dynamic analysis of B cell numbers showed that there was a transient decrease of B-1 cell numbers in PEC after *C. albicans* inoculation, and then the numbers increased and peaked at 36h after inoculation (data not shown). The subsets of B-1 cells were analyzed at 36 h after inoculation as shown in Fig. 3. The number of CD5^+^ B-1a cells in TgV_H_3B4 was higher than that of littermate before *C. albicans* inoculation (Fig. 3), which has been reported in our previous study [8]. While after *C. albicans* inoculation, numbers of B-1a cells increased in both TgV_H_3B4 and littermate, and TgV_H_3B4 has higher number of B-1a cells.

**Figure 4:**
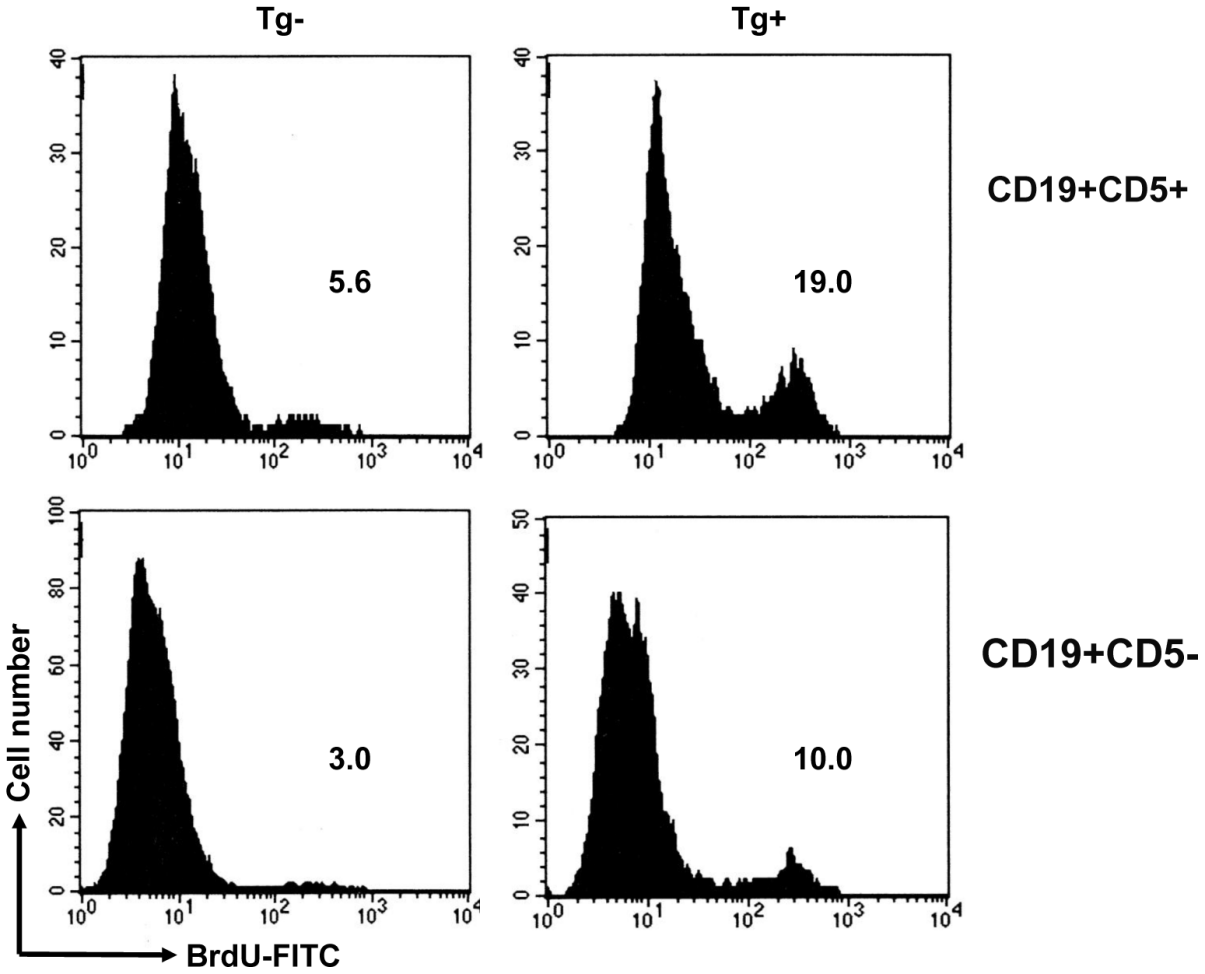
BrdU incorporation assay. BrdU was i. p. injected with *C. albicans* inoculation to TgV_H_3B4 mice and littermate, and injected for the second time 12 h later. PEC cells were eluted at 36 h after inoculation and BrdU labeling was performed using a BrdU Labeling kit and analyzed by FCM. Data are representative of four independent experiments.

The increase in B-1a cell number may be the result of enhanced proliferation or accelerated migration. Consequently, we further assessed the proliferation of B-1 cells by using BrdU incorporation assay. Results showed that before PEC inoculation there was only a detectable incorporation of BrdU in B-1a and B-1b cells in both TgV_H_3B4 and control mice (data not shown). After PEC *C. albicans* inoculation, there were marked proliferation of both B-1a and B-1b cells, as shown by 19% and 10% BrdU^+^ cells (Fig. 4). The proliferation of B-1a cells in TgV_H_3B4 was greater than that of B-1b cells, indicating that B-1a cells responded more actively in immediate immunity to *C. albicans* infection.

### Clonal expansion of *C. albicans*-specific B cells in TgVH3B4

**Figure 5:**
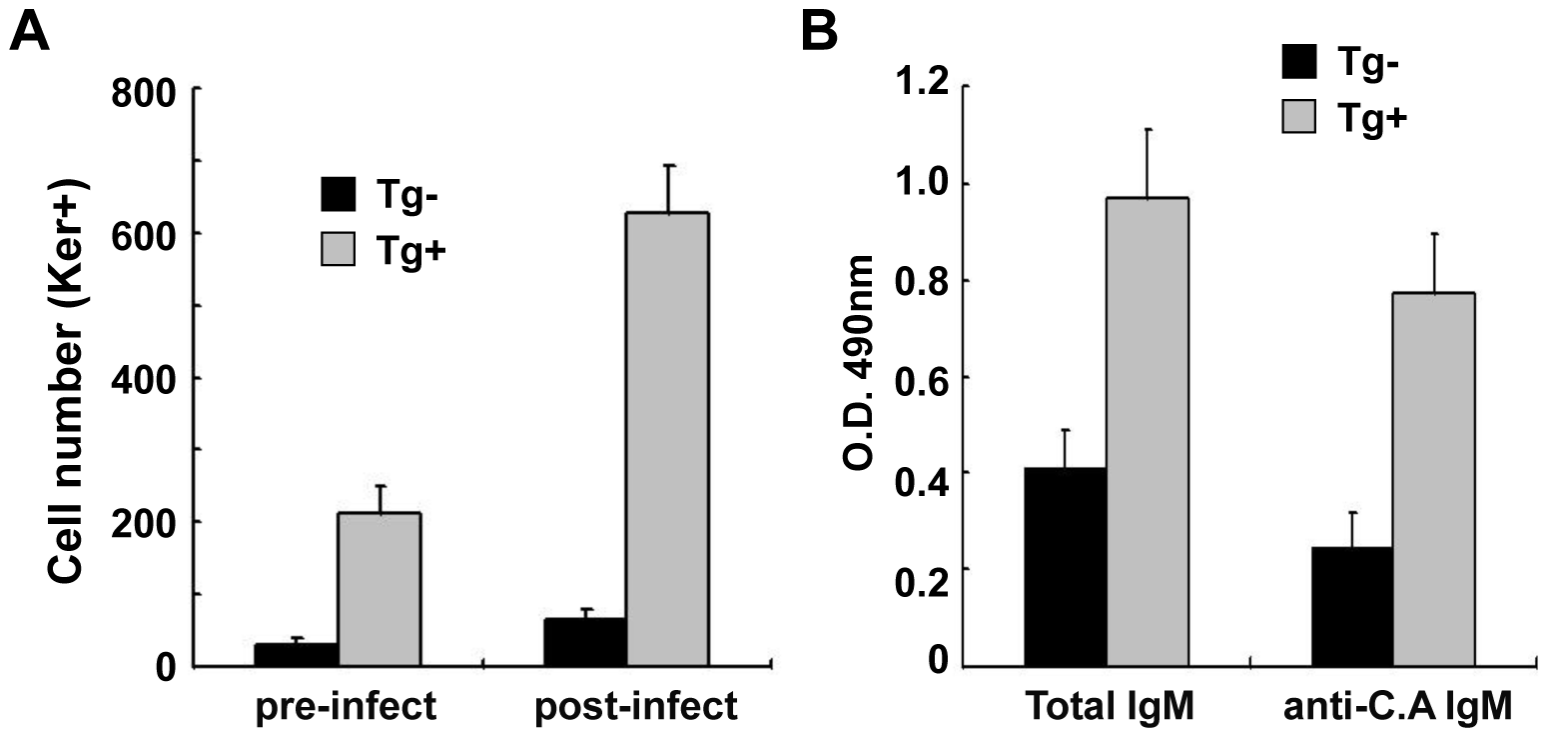
Analysis of *C. albicans*-specific B cells. (A) Cells collected from the PEC lavage before and after *C. albicans* inoculation were subjected to ELISPOT analysis using plate coated with mouse keratin. (B) Supernatant from the PEC B cells co-cultured with *C. albicans* were analyzed by ELISA to detect total IgM and *C. albicans*-specific IgM. Data shown are means±SD, n = 6. * *P*<0.01.

To determine the antigen-specificity of the proliferating B cells after *C. albicans* inoculation in PEC, we sorted B cells by magnetic beads from PEC, and analyzed by using ELISPOT assay with keratin as coating antigen [8], as polyreactive natural antibody 3B4 can identify both *C. albicans* and keratin. Before *C. albicans* inoculation, there were more keratin reactive B cells in PEC of TgV_H_3B4 mice than control, which has been shown in previous study [8], [26]. After i. p. *C. albicans* inoculation, there were even more B cells secreting keratin-reactive IgM in TgV_H_3B4 mice (Fig. 5A). In the PEC of littermate control, there were also more keratin-reactive B cells after fungal inoculation, but the increasing scope was very limited (Fig. 5A). When B cells from PEC were co-cultured with irradiated *C. albicans*, both total IgM and *C. albicans*-specific IgM in the supernatant were much higher in TgV_H_3B4 mice than that of control (Fig. 5B). These data indicated that there was active clonal expansion of *C. albicans*-specific B-1 cells in TgV_H_3B4 mice after infection with *C. albicans*.

## Discussion

B-1 cells reside predominantly in the PEC, bearing several features of activated B cells, and act as key players in the early humoral response against pathogens by producing natural antibodies [16]. From an evolutionary point of view, it is interesting to note that B-1 cells may represent a transitional phase on the evolution of the adaptive immune response, guarding the main compartments of the primitive organism, the body cavity [27]. One function of peritoneal B1 cells is to survey the abdominal cavity and to join forces with innate cells, such as macrophages, for a rapid and efficient pathogen clearance. *C. albicans* is the main commensal organism of the gastrointestinal tract, and it is reasonable to speculate that B-1 cells might have a role against *C. albicans* infection. In the past several years, it has been reported that B-1 cells play an important role in host defense against many pathogens including bacteria [4], [23], viruses [6] and lymphatic filarial parasites [28]. However, the role of B-1 cells in fungi infection remains unclear. Using a monoclonal antibody 3B4 and TgV_H_3B4 mice with V_H_ derived from 3B4, we have previously demonstrated a key role of natural antibodies in immune responses against *C. albicans* infection [8]. In this study, we explored the role and responses of B-1 cells to *C. albicans* infection by using i. p. fungi inoculation. We found that TgV_H_3B4 mice cleared the peritoneal *C. albicans* infection more efficiently, and level of *C. albicans*-reactive IgM in the PEC of TgV_H_3B4 mice was higher than that of control. Number of B-1a cells was also increased, which was mainly due to enhanced B-1 cell proliferation. Furthermore, *C. albicans*-specific B cells increased numerously in TgV_H_3B4 mice after *C. albicans* infection. These data suggested that upon peritoneal *C. albicans* infection, *C. albicans*-specific B-1 cells proliferate actively and produce a large amount of natural IgM to clear the pathogen. It also indicated that B-1 cells play a critical role in host defense against fungal infection.

One of the most important functions of B-1 cells is producing natural antibodies; however, where B-1 cells produce natural antibodies is not fully understood. Studies showed that PEC B cells are precursors of splenic IgM natural antibody-producing cells and IgA producing plasma cells in murine gut [29], [30]. Recently, Fagarasan et al found that antibody production by B-1 cells is closely related with their migration capacity to other lymphoid organs [31]; Wen et al showed that B-1 cells migrate to spleen and produce antibodies [32]. In our study, however, there was anti-*C. albicans* natural antibodies exist in the PEC of TgV_H_3B4 and littermate, and TgV_H_3B4 mice had much higher level of *C. albicans*-specific natural antibodies, indicating that B-1 cells can also produce natural antibodies in PEC without the need for migration.

After *C. albicans* inoculation in PEC, there was a rapid increase of *C. albicans*-specific IgM and *C. albicans*-specific B cells, indicating that the pre-existing *C. albicans*-reactive B-1 cells in PEC acted actively in early defense against infection. Clonal expansion of B-1 cells after infection has been discussed in several literatures; however, the data from different studies are inconsistent with one other. Baumgarth et al found that the protection against influenza virus infection by B-1 cells-derived IgM antibodies does not entail significant increases in their normal serum levels after infection [33], suggesting that clonal expansion and increased IgM secretion is not initiated *in vivo* after infection. In Matin's paper, however, T15 idiotype expressing B-1 cells expanded vigorously in both PEC and spleen [25]. Our results are in consistent with Martin's study: *C. albicans*-specific B-1 cells proliferate in the PEC, and produce a high amount of natural antibody to clear *C. albicans* infection. The absence of B-1 cell expansion in Baumgarth's work may be due to a different route of infection: blood steam transmitted virus may not have the chance to meet B-1 cells in the spleen and PEC. While in our and Matin's experiment, i. p. infection were applied, and B-1 cells could contact with the pathogen and were stimulated to proliferate and produce antibody. Besides natural antibodies and B-1 cells, there are many other components of innate immunity against *C. albicans* infection in PEC, including epithelial cells, macrophages, neutrophils, NK cells, complement, and so on. Mechanisms of cooperation between different cells remain to be further investigated in the future.

As the transgene used to construct the TgV_H_3B4 mice contains only the IgH μ chain; consequently, the effect of other isotypes of immunoglobulin such as IgG cannot be explored using our system. The infection in our model was limited in the PEC, and the role of B-1 cells in disseminated *C. albicans* infection cannot be explored. More studies are needed to further our understanding on the role of B-1 cells in host defense against fungi infection.

## Materials and Methods

### Ethics Statements

The animal husbandry, experiments and welfare were conducted in accordance with the Detailed Rules for the Administration of Animal Experiments for Medical Research Purposes issued by the Ministry of Health of China, and were approved by the Animal Experiment Administration Committee of Fourth Military Medical University. Mice were raised in the specific pathogen free conditions on the C57BL/6 background, and were manipulated with every specific care to reduce the suffering of the mice during the experiments.

### Mice

Transgenic mice with V_H_ derived from a monoclonal antibody 3B4 were generated by our group as described previously [8]. The mice were housed in special pathogen free conditions, and backcrossed with C57BL/6 mice for more than eight generations before analysis. All animal protocols were approved by the University's Animal Research Committee.

### Fungal inoculation and PEC lavage preparation

*C. albicans* (ATCC 90028) were cultured in the Sabouraud's dextrose medium. For *in vivo* inoculation, 2×10^6^ *C. albicans* yeasts were injected i. p. into 6∼8-week-old TgV_H_3B4 mice and littermate control. At different time point after inoculation (6 h, 12 h, 24 h, 36 h) the mice were sacrificed. PEC lavage was eluted as described previously. In brief, a small hole was cut by scissors on the abdomen wall of the mice, and 1 ml PBS was injected into the PEC with a pipette. Then a pasture tube was used to collect the cell suspension from the PEC. The same procedure was repeated three times and about 3.5 ml lavage from the PEC can be harvested. The supernatant of the first 1 ml of elution was saved for cytokine analysis, and the cells from all the three elution were pooled and subjected to flow cytometry (FCM) analysis and colony-forming assay immediately. Three mice were included in one group and each experiment was repeated for at least three times.

### Colony-forming assay and cytokine detection

Living yeasts were revealed by a colony-forming assay. In brief, the elution were spread onto an agar plate and cultured at 37°C overnight, and then the colonies were counted. Cytokines in the elution were analyzed by using a Cytometry Beads Array kit (BD Bioscience, San Diego, CA) according to the manufactures instruction.

### ELISA

To measure *C. albicans*-reactive IgM, *C. albicans* yeasts were seeded in microtitre plates (Nunc, Roskilde, Denmark) at a density of 1×10^5^/100 µl in RPMI 1640 and cultured at 37°C for 2 h to let yeast cells adhere tightly onto the plates, as described previously [8]. For analysis of mouse IgM, goat anti-mouse μ chain antibody (Sigma-Aldrich) was coated to microtitre plates at a concentration of 1 µg/ml. PEC lavage from *C. albicans* inoculated TgV_H_3B4 and control mice or supernatant of cell culture were added and incubated at room temperature for 1 h. After washing, the biotin-labeled anti-IgM (Sigma-Aldrich) was added and incubated, followed by incubation with the streptavidin-horseradish peroxidase, and developed by O-phenylenediamine.

### Flow cytometry

Cells collected from mouse PEC lavage were stained with antibodies for 30 min on ice, and analyzed by using a FACSCalibur™ (BD Immunocytometry Systems, San Jose, CA). Dead cells were excluded by propidium iodide staining. Data were analyzed by using the Cellquest™ software. Antibodies used in analyses included: anti-CD14 (rmC5-3), anti-Gr-1 (RB6-8C5), anti-CD5 (53–7.3), anti-CD19 (1D3), anti-Mac-1 (M1/70). All the antibodies were purchased from BD PharMingen (San Diego, CA).

### BrdU incorporation

0.6mg (200 µL/mouse) BrdU (5-Bromo-2-deoxyuridine) (Sigma-Aldrich) was i. p. injected with fungal inoculation to TgV_H_3B4 mice and littermate control, and was injected for the second time 12 h later. After 36 h of inoculation, cells from PEC of mice were subjected for BrdU staining, which was performed using a BrdU Labeling kit (BD PharMingen, San Diego, CA) according to the manufacturer's instructions. Cells were then analyzed by FCM.

### Enzyme-linked immunoSPOT (ELISPOT) assay

PEC B cells were sorted by first staining with anti-mouse CD19-biotin (BD Biosciences) and then by BD IMag magnetic beads conjugated with avidin (BD Biosciences) according to the manufacturer's instructions. Purified B cells were plated at 2×10^5^ cells/well in 96-well plates pre-coated with 20 µg/ml of purified mouse epidermis keratin, as the 3B4 antibody can also bind keratin. Cells were stimulated with LPS (50 µg/ml, Sigma-Aldrich) for 5 days at 37°C in an atmosphere of 5% CO_2_. Wells were washed thoroughly with PBS containing 0.02% Tween-20, and the biotin-labeled anti-IgM was added. Plates were incubated for 2 h at room temperature, washed, and incubated with the streptavidin-alkaline phosphotase (Jackson Immunoresearch, Boston, MA) for 75 min at room temperature. Spots were developed by addition of the BCIP substrate (Sigma-Aldrich), and the reaction was stopped by washing extensively with water. Wells were counted under a microscope.

### In vitro cell culture

PEC B cells were sorted as described above. The sorted cells were plated at a density of 1×10^5^ cells/well in 96-well plates. γ-irradiated *C. albicans* yeasts were seeded in the well at 5×10^5^ cells/well and co-cultured with PEC B cells. After 3 days, the supernatant of culture was collected and subjected to ELSA analysis of total IgM and *C. albicans*-specific IgM.

### Statistics

ANOVA was used to determine the statistical significance of values among experimental groups. Statistical significance was defined as p<0.05.

## References

[1] Boes M (2000) Role of natural and immune IgM antibodies in immune responses. Mol Immunol 37: 1141–1149. 11451419 10.1016/s0161-5890(01)00025-6

[2] Quintana FJ, Cohen IR (2004) The natural autoantibody repertoire and autoimmune disease. Biomed Pharmacother 58: 276–281. 15194162 10.1016/j.biopha.2004.04.011

[3] Belperron AA, Bockenstedt LK (2001) Natural antibody affects survival of the spirochete Borrelia burgdorferi within feeding ticks. Infect Immun 69: 6456–6462. 11553590 10.1128/IAI.69.10.6456-6462.2001 PMC98781

[4] Boes M, Prodeus AP, Schmidt T, Carroll MC, Chen J (1998) A ritical role of natural immunoglobulin M in immediate defense against systemic bacterial infection. J Exp Med 188: 2381–2386. 9858525 10.1084/jem.188.12.2381 PMC2212438

[5] Lepper PM, Moricke A, Held TK, Schneider EM, Trautmann M (2003) K-antigen-specific, but not O-antigen-specific natural human serum antibodies promote phagocytosis of Klebsiella pneumoniae. FEMS Immunol Med Microbiol 35: 93–98. 12628543 10.1016/S0928-8244(02)00459-5

[6] Ochsenbein AF, Fehr T, Lutz C, Suter M, Brombacher F, et al (1999) Control of early viral and bacterial distribution and disease by natual antibodies. Science 286: 2156–2159. 10591647 10.1126/science.286.5447.2156

[7] Shibuya A, Sakamoto N, Shimizu Y, Shibuya K, Osawa M, et al (2000) Fc α/μ receptor mediates endocytosis of IgM-coated microbes. Nat Immunol 1: 441–446. 11062505 10.1038/80886

[8] Li W, Fu M, An JG, Xing Y, Zhang P, et al (2007) Host defence against C. albicans infections in IgH transgenic mice with V(H) derived from a natural anti-keratin antibody. Cell Microbiol 9: 306–315. 16925788 10.1111/j.1462-5822.2006.00786.x

[9] Taborda CP, Casadevall A (2002) CR3 (CD11b/CD18) and CR4 (CD11c/CD18) are involved in complement-independent antibody-mediated phagocytosis of cryptococcus neoformans. Immunity 16: 791–802. 12121661 10.1016/s1074-7613(02)00328-x

[10] Zhang MX, Bohlman MC, Itatani C, Burton DR, Parren PW, et al (2006) Human recombinant antimannan immunoglobulin G1 antibody confers resistance to hematogenously disseminated candidiasis in mice. Infect Immun74: 362–369. 10.1128/IAI.74.1.362-369.2006 PMC1346657 16368991

[11] Maiti PK, Kumar A, Kumar R, Mohapatra LN (1985) Role of antibodies and effect of BCG vaccination in experimental candidiasis in mice. Mycopathologia 91: 79–85. 3900732 10.1007/BF00436540

[12] Wagner RD, Vazquez-Torres A, Jones-Carson J, Warner T, Balish E (1996) B cell knockout mice are resistant to mucosal and systemic candidiasis of endogenous origin but susceptible to experimental systemic candidiasis. J Infect Dis 174: 589–597. 8769618 10.1093/infdis/174.3.589

[13] Kozel TR, MacGill RS, Percival A, Zhou Q (2004) Biological activities of naturally occurring antibodies reactive with Candida albicans mannan. Infect Immun 72: 209–218. 14688098 10.1128/IAI.72.1.209-218.2004 PMC343987

[14] Zhang MX, Lupan DM, Kozel TR (1997) Mannan-specific immunoglobulin G antibodies in normal human serum mediate classical pathway initiation of C3 binding to Candida albicans. Infect Immun 65: 3822–3827. 9284158 10.1128/iai.65.9.3822-3827.1997 PMC175545

[15] Fu M, Fan PS, Li W, Li CX, Xing Y, et al (2007) Identification of poly-reactive natural IgM antibody that recognizes late apoptotic cells and promotes phagocytosis of the cells. Apoptosis 12: 355–362. 17191117 10.1007/s10495-006-0581-z

[16] Berland R, Wortis HH (2002) Origins and functions of B-1 cells with notes on the role of CD5. Annu Rev Immunol 20: 253–300. 11861604 10.1146/annurev.immunol.20.100301.064833

[17] Hardy RR, Hayakawa K (2001) B cell development pathways. Annu Rev Immuno 19: 595–621. 10.1146/annurev.immunol.19.1.595 11244048

[18] Hayakawa K, Asano M, Shinton SA, Gui M, Allman D, et al (1999) Positive selection of natural autoreactive B cells. Science 285: 113–116. 10390361 10.1126/science.285.5424.113

[19] Hayakawa K, Asano M, Shinton SA, Gui M, Wen LJ, et al (2003) Positive selection of anti-thy-1 autoreactive B-1 cells and natural serum autoantibody production independent from bone marrow B cell development. J Exp Med 197: 87–99. 12515816 10.1084/jem.20021459 PMC2193793

[20] Binder CJ, Shaw PX, Chang MK, Boullier A, Hartvigsen K, et al (2005) The role of natural antibodies in atherogenesis. J Lipid Res 46: 1353–1363. 15897601 10.1194/jlr.R500005-JLR200

[21] Rowley B, Tang L, Shinton S, Hayakawa K, Hardy RR (2007) Autoreactive B-1 cells: constraints on natural autoantibody B cell antigen receptors. J Autoimmun 29: 236–245. 17889506 10.1016/j.jaut.2007.07.020 PMC2096705

[22] Zhang M Jr, Austen WG, Chiu I, Alicot EM, Hung R, et al (2004) Identification of a specific self-reactive IgM antibody that initiates intestinal ischemia/reperfusion injury. Proc Natl Acad Sci USA 101: 3886–3891. 14999103 10.1073/pnas.0400347101 PMC374339

[23] Haas KM, Poe JC, Steeber DA, Tedder TF (2005) B-1a and B-1b cells exhibit distinct developmental requirements and have unique functional roles in innate and adaptive immunity to S. pneumoniae. Immunity 23: 7–18. 16039575 10.1016/j.immuni.2005.04.011

[24] Martin F, Kearney JF (2000) Positive selection from newly formed to marginal zone B cells depends on the rate of clonal production, CD19, and btk. Immunity 12: 39–49. 10661404 10.1016/s1074-7613(00)80157-0

[25] Martin F, Oliver AM, Kearney JF (2001) Marginal zone and B-1 B B cells unite in the early response against T-independent blood-borne particulate antigens. Immunity 14: 617–629. 11371363 10.1016/s1074-7613(01)00129-7

[26] Xing Y, Li W, Lin Y, Fu M, Li CX, et al (2009) The influence of BCR density on the differentiation of natural poly-reactive B cells begins at an early stage of B cell development. Mol Immunol 46: 1120–1128. 19136153 10.1016/j.molimm.2008.10.031

[27] Janeway CA (2001) Immunobiology. 5th edition. New York: Garland Pub.

[28] Paciorkowski N, Porte P, Shultz LD, Rajan TV (2000) B-1 B lymphocytes play a critical role in host protection against lymphatic filarial parasites. J Exp Med 191: 731–736. 10684864 10.1084/jem.191.4.731 PMC2195839

[29] Kawahara T, Ohdan H, Zhao G, Yang YG, Sykes M (2003) Peritoneal cavity B cells are precursors of splenic IgM natural antibody-producing cells. J Immunol 171: 5406–5414. 14607944 10.4049/jimmunol.171.10.5406

[30] Kroese FG, Butcher EC, Stall AM, Lalor PA, Adams S, et al (1989) Many of the IgA producing plasma cells in murine gut are derived from self-replenishing precursors in the peritoneal cavity. Int Immunol 1: 75–84. 2487677 10.1093/intimm/1.1.75

[31] Fagarasan S, Shinkura R, Kamata T, Nogaki F, Ikuta K, et al (2000) Alymphoplasia (aly)-type nuclear factor kappaB-inducing kinase (NIK) causes defects in secondary lymphoid tissue chemokine receptor signaling and homing of peritoneal cells to the gut-associated lymphatic tissue system. J Exp Med 191: 1477–1486. 10790423 10.1084/jem.191.9.1477 PMC2213441

[32] Wen L, Shinton SA, Hardy RR, Hayakawa K (2005) Association of B-1 cells with follicular dendritic cells in spleen. J Immunol 174: 6918–6926. 15905534 10.4049/jimmunol.174.11.6918

[33] Baumgarth N, Herman OC, Jager GC, Brown LE, Herzenberg LA, et al (2000) B-1 and B-2 cell-derived immunoglobulin M antibodies are nonredundant components of the protective response to influenza virus infection. J Exp Med 192: 271–280. 10899913 10.1084/jem.192.2.271 PMC2193249

