# Correction: In Situ IgM Production and Clonal Expansion of B-1 Cells in Peritoneal Cavity Promote Elimination of *C. albicans* Infection in IgH Transgenic Mice with V_H_ Derived from a Natural Antibody

**DOI:** 10.1371/annotation/bedab014-d393-4fad-aa07-0df1efb59038

**Published:** 2013-10-09

**Authors:** Rong Tian, Meng Fu, Zhuo Zhang, Jing Ren, Jingang An, Yufeng Liu, Wei Li

Affiliation number 2 for the first, second, fourth, fifth, sixth and seventh authors is incorrect. The correct affiliation is: Department of Dermatology, Xijing Hospital, Fourth Military Medical University, Xi’an, P. R. China. 

