# Correction: In Situ IgM Production and Clonal Expansion of B-1 Cells in Peritoneal Cavity Promote Elimination of *C. albicans* Infection in IgH Transgenic Mice with VH Derived from a Natural Antibody

**DOI:** 10.1371/journal.pone.0118400

**Published:** 2015-03-05

**Authors:** The PLOS ONE Staff

The article has been reviewed by the academic committee of the Department of Dermatology at Xijing Hospital, Fourth Military Medical University which considered that Rong Tian and Zhuo Zhang should not have been included in the author list. In line with the recommendation issued by the academic committee of the Department of Dermatology at Xijing Hospital, the authors would like to make the following changes to the author list:

Rong Tian was incorrectly listed as an author on the article, she completed preliminary work for this study however the data generated from those experiments were not included in the final article. Zhuo Zhang provided some reagents for this study but his contribution does not fulfil the authorship criteria.

The authors apologize for the inaccuracies in the original author list. The correct author list is:

Meng Fu^1^, Jing Ren^1^, Jingang An^1^, Yufeng Liu^1^, Wei Li^1,2^

Affiliations:

1. Department of Dermatology, Xijing Hospital, Xi’an, P. R. China

2. Department of Dermatology, General Hospital of the Air Force, Beijing, P. R. China

The summary of contributions to the study should be revised as below:

Conceived and designed the experiments: WL YL.

Performed the experiments: MF JR JA.

Analyzed the data: WL YL.

Wrote the paper: WL YL.

Rong Tian and Zhuo Zhang are acknowledged for their involvement with this work, the Acknowledgements section would be revised as follows:

We thank Hua Han for his suggestion and Demirjian Marine for her critical reading and improvement of writing. We also thank Rong Tian for contribution to early stages of the project and Zhuo Zhang for the provision of reagents.
